# Experimental Study on the High Temperature Impact Torsional Behavior of Ti-1023 Alloy

**DOI:** 10.3390/ma15113847

**Published:** 2022-05-27

**Authors:** Lintao Li, Zhihua Wang, Wei Ma

**Affiliations:** 1 Institute of Applied Mechanics, College of Mechanical and Vehicle Engineering, Taiyuan University of Technology, Taiyuan 030024, China; lintao_tyut@163.com; 2 Shanxi Key Laboratory of Material Strength and Structural Impact, Taiyuan University of Technology, Taiyuan 030024, China; 3 Institute of Mechanics, Chinese Academy of Sciences, Beijing 100190, China; watwm@imech.ac.cn

**Keywords:** high temperature, dynamic torsion, titanium alloy, stress-induced martensite

## Abstract

Based on the modified Hopkinson torsion bar, a high-temperature dynamic shear test method was proposed for the Ti-1023 alloy, and the microstructural evolution of the tested material at different temperatures was studied. By using the modified high-temperature Hopkinson torsion bar, high-temperature testing within 1000 °C can be achieved. As the specimen-heating rate was fast, and the temperature gradient of the experimental environment was small, valid experimental data can be ensured during the experiment. The experimental results show that stress-induced martensite can significantly enhance the strength of the Ti-1023 alloy. Dynamically recrystallized grains similar to those in adiabatic shear bands appear in the microstructure of the Ti-1023 alloy after severe plastic deformation. Therefore, it is possible to regulate the content of stress-induced martensite in the microstructure to improve the mechanical properties of other alloys that are similar to β titanium alloys.

## 1. Introduction

Dynamic mechanical properties are an important index parameter for material service. Under high-strain-rate-loading conditions, the yield stress and failure mechanism of materials are significantly different from those under quasi-static loading conditions [1,2]. Most engineering materials have a strain-rate-sensitive effect during service. The study of the strain-rate effect of materials is not only of theoretical significance but also of practical value in engineering applications. At present, the dynamic behavior of materials is usually studied by using Hopkinson experimental techniques, such as the split Hopkinson pressure bar (SHPB) and Hopkinson tension bar experimental equipment [3,4]. Such an experimental technique is mainly based on the assumption of a one-dimensional stress wave and the assumption of uniform deformation. However, the elastic wave has a dispersion phenomenon during the propagation of the guide bar, and there is friction between the specimen and the end of the bar. These problems have a direct effect on the experimental accuracy [5,6]. The split Hopkinson torsion bar is the development of the SHPB experimental technology. Compared with the Hopkinson pressure bar, the torsional wave has no dispersion phenomenon during the propagation process, and the specimen has no radial motion; thus, there is no interface friction between the specimen and the guide bar, which is helpful to improving the experimental accuracy [7,8]. A lathe-based Hopkinson torsion bar was first proposed by Baker and Yew et al. [9]. Since then, the Hopkinson torsion bar has been widely used. Clyens et al. [10] used the Hopkinson torsion bar to measure the viscosity of supercooled liquids at high-shear rates. Bassim et al. [11] experimentally tested high-strain-rate shear properties of a high-strength steel (Algtuff 400F). Duffy et al. [12,13,14] studied the high-strain-rate-shear behavior of 1100 aluminum alloy and HY-100 structural steel by improving the Hopkinson torsion bar. The results showed that the plastic deformation followed a three-stage process, with the first a uniform-strain state, then a general inhomogeneous-strain distribution, and finally a localized reduction to a thin-shear band. Yang et al. [15] performed dynamic torsion tests at a strain-rate level of 10^3^ on L4 pure aluminum and 20# steel on a self-made torsion bar and found that a larger deformation could be achieved by using the Hopkinson torsion bar than the Hopkinson compression bar at the same strain rate. Chen et al. [16] obtained a gradient microstructure by performing multiple torsional shocks on high-entropy alloys, thereby improving the properties of the tested alloys. Jiang et al. [17,18] and Li et al. [19] modified the Hopkinson torsion bar and developed a T-shaped torsion bar device, an electromagnetic reluctance type Hopkinson torsion bar, and a hydraulic embracing locking and releasing device, which further enriched the Hopkinson torsion bar experiment technique.

The mechanical behavior of materials under a high-temperature environment is also attracting the interest of some scholars. In quasi-static high-temperature experiments, the high-temperature environment is generally achieved by sealing the specimen and the loading fixture together in a high-temperature environment box and then heating for a long time. However, the technology of realizing a high-temperature environment in quasi-static material experiments cannot be easily extended to dynamic experiments. The main reason is that the long-term heating of the dynamic experimental equipment will lead to changes in its wave impedance so that the experimental results cannot accurately reflect the properties of the tested materials [20]. At present, the dynamic test under the high-temperature environment generally adopts the preheating of specimens, and then the test is carried out after the bar is butted. This method is mainly used for Hopkinson compression bars [21,22,23]. However, the Hopkinson torsion bar that can perform high-temperature experiments has been rarely reported.

The dynamic mechanical properties of titanium alloys have been extensively investigated. Khan et al. [24] investigated the mechanical properties of Ti6Al4V over wide ranges of strain rates and temperatures based on the Hopkinson pressure bar. Ranc, N. et al. [25] used the Hopkinson torsion bar to study the damage and failure mechanisms of titanium alloys under dynamic loading. The initiation and propagation mechanisms of ASB were investigated by measuring the temperature distribution of the adiabatic shear band. There are few studies on dynamic pure shearing of titanium alloys at high temperatures.

In this paper, the dynamic shear test under the high-temperature environment will be realized by improving the Hopkinson torsion bar, and the high-temperature dynamic shear test of Ti-1023 alloy will be carried out.

## 2. Materials and Methods

### 2.1. Modified Hopkinson Torsion Bar

**Figure 1:**
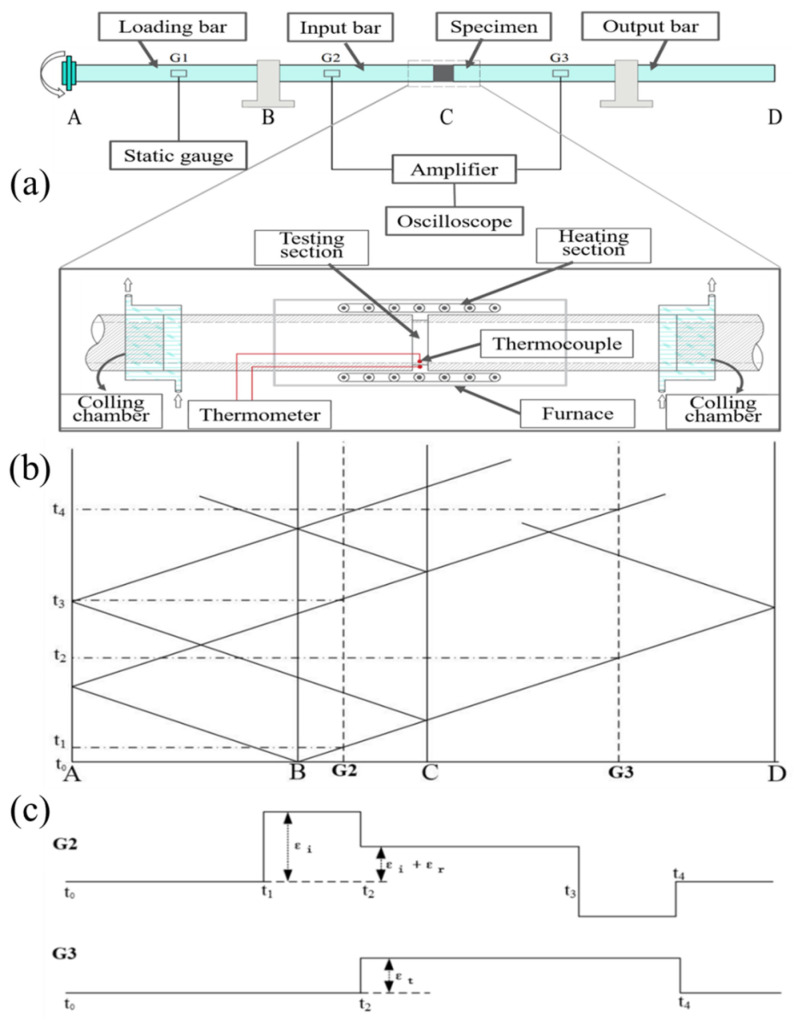
Hopkinson torsion bar experimental device schematic diagram (**a**) and curves of torsional wave propagation and ideal waveforms with constant specimen stiffness: (**b**) wave propagation characteristic line, (**c**) ideal waveform.

The schematic diagram of the high-temperature Hopkinson torsion bar experimental device is shown in Figure 1a. Similar to the Hopkinson pressure bar, the experimental device consists of an incident bar, a transmission bar, a torque-loading chuck, and a heating and cooling system. The waveguide bars, AC and CD, are the incident bar and the transmission bar, respectively, and B is the locking mechanism on the incident bar, which is used to lock the incident bar to store torque. The thin-walled round tube specimen is glued at C by super glue. G1, G2, and G3 are the strain rosettes for recording data, and G1 is used to record the static-loading torque to ensure that the incident bar is always within the elastic limit. G2 and G3 are rosettes used to record input and output waveforms. To realize the high-temperature test, it was modified on the basis of the original equipment, and a high-temperature chamber and a water-cooling chamber were added, respectively (Figure 1, the enlarged image at C). Before the start of the experiment, the locking mechanism B was locked with a bolt with a prefabricated cutout to lock the incident bar to store the torque, and the loading plate A was rotated by the hydraulic jack to generate the torque. During the experiment, with screwing off the bolt at the locking mechanism B, the instant release of the locking chuck will generate the torsional pulse wave required for the experiment. According to the propagation characteristics of the torsional wave in the waveguide bar, its characteristic line is drawn, as shown in Figure 1b. The torsional wave is generated when the buckle is released and instantaneously propagates to both ends at the locking mechanism B. The right traveling wave passes through the rosette G2 at the time t_1_ and reaches the specimen C for reflection and transmission. The transmitted wave continues to propagate to the right through the rosette G3 at time t_2_, and the reflected wave generated at the specimen C propagates to the left through G2. The rosettes G2 and G3 record the incidents of the reflected and transmitted waves, respectively. Under ideal conditions when the stiffness of the specimen is assumed to be constant and the strain rosette can receive rectangular waves, the characteristic line of its wave amplitude is drawn, as shown in Figure 1c, where I, R, and T represent the amplitude of the incident wave, the reflected wave, and the transmission, respectively.

**Figure 2:**
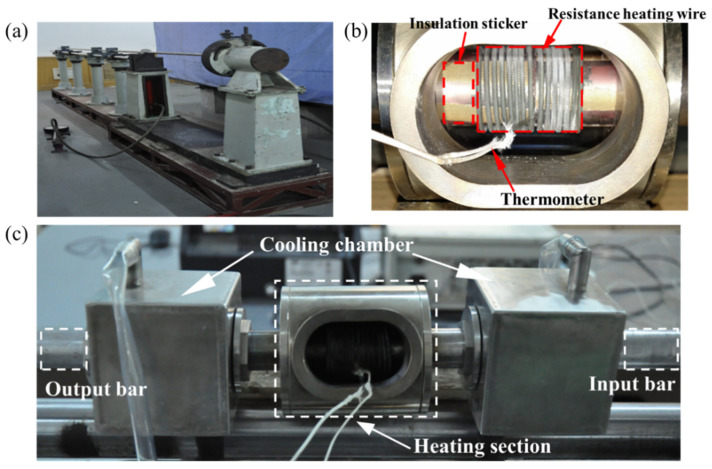
Modified Hopkinson torsion bar experimental device: (**a**) experimental device physical map; (**b**) specimen and high greenhouse assembly diagram; (**c**) high-temperature cooling system.

The physical map of the experimental device and the state of the specimen in the high-temperature chamber are shown in Figure 2. The high-temperature chamber uses a spring-like Cr20Ni80 resistance-heating wire to directly heat the specimen. When the specimen is assembled, it passes through the high-temperature chamber and the heating wire, and the temperature of the experimental section of the specimen is monitored in real time using a thermocouple wire. It should be noted that insulation treatment should be done between the specimen, the high-temperature chamber, and the thermocouple wire. To prevent the failure of the strain, roses are pasted on the waveguide bar due to short circuiting during the experiment. Therefore, the specimen needs to be pasted with a high-temperature insulating sticker on the part of the surface in contact with the resistance-heating wire before assembly (see Figure 2b). Similarly, the front end of the thermocouple wire is also affixed with the same sticker. During the experiment, attention should also be paid to sealing the place where the cooling chamber is in contact with the specimen and the waveguide bar (see Figure 2c). Since the cooling of the guide bar is carried out by using circulating water, once the seal fails, it will also cause problems, such as short circuit of the heating wire, resulting in the failure of the experiment.

The heating furnace used in the experiment is made of nickel-based super alloy and can withstand temperatures up to 1500 °C. At the same time, the furnace body is covered with an asbestos interlayer to reduce heat loss and to shorten the heating time. The insulation treatment should be done during the heating process to prevent the short circuit of resistance strain rosettes. Two cooling chambers are respectively placed at the bonding interface between the waveguide bar and the specimen. Circulating water is used for cooling to ensure that the adhesive will not be carbonized and degummed and that the waveguide bar will not be directly heated.

**Figure 3:**
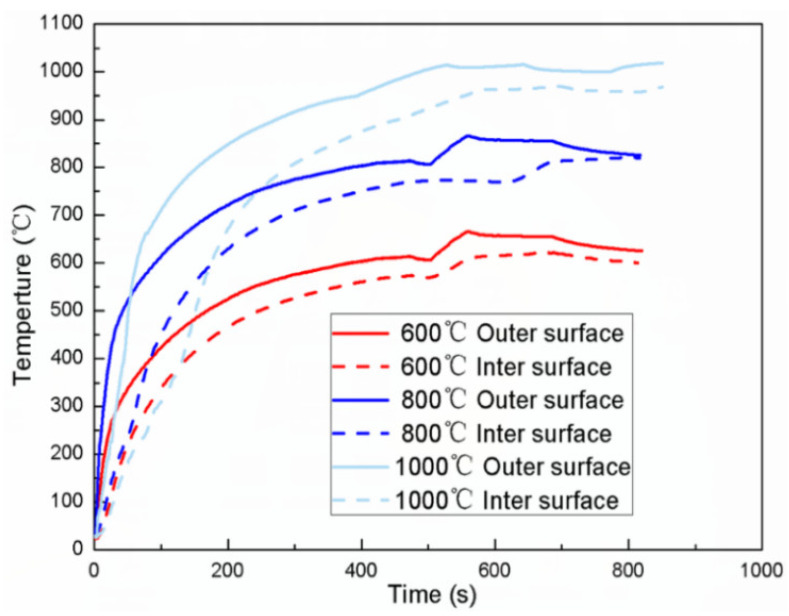
Temperature change of inner and outer walls of the specimen during heating.

In the high-temperature experiment, the specimen must be in a uniform-temperature field to ensure the reliability of the experimental data. During the heating process, the temperatures of the inner and outer walls of the specimen were monitored using a thermocouple wire. Figure 3 shows the temperature curves of the inner and outer walls of the specimen at 600, 800, and 1000 °C. The results show that the temperature difference between the inner and outer walls of the specimen can be controlled within 5% during the experiment, and a uniform high-temperature environment can be achieved.

Signal processing is derived from the strain flow-recorded strain based on the one-dimensional stress wave theory. When the dynamic stress balance is reached inside the specimen, the stress, strain, and strain rate of the material can be expressed as:

(1) $$\tau =G_{b}\frac{r_{s}J_{b}}{r_{b}J_{s}}\varepsilon_{t}\;\;\;\;\gamma =2\frac{r_{s}C}{r_{b}l}\int \varepsilon_{r}dt\;\;\;\;\dot{\gamma }=2\frac{r_{s}C}{r_{b}l}\varepsilon_{r}$$

where *G* is the shear modulus of elasticity of the incident and transmitted bar, *C* is the elastic wave velocity in the waveguide bar, *r_s_* and *r_b_* are the average radius of the thin-walled tube specimen and the radius of the waveguide bar, respectively, *J* is the polar moment of inertia, and *ε_t_(t), ε_r_(t)* are the transmitted and reflected wave-strain signals, respectively. Through the above formula, the strain rate and shear stress–strain relationship of the specimen under dynamic-loading conditions can be obtained.

### 2.2. Experimental Material

As-cast Ti-1023 titanium alloy provided by the Northwest Nonferrous Metals Research Institute was used as the experimental material, and its chemical compositions are shown in Table 1. Some key performance indicators of the studied material are as follows: the density is 4.62 g/cm^3^, the melting point is 1800 °C, and the yield strength and ultimate tensile strength are 1030 and 1105 MPa, respectively.

**Figure 4:**
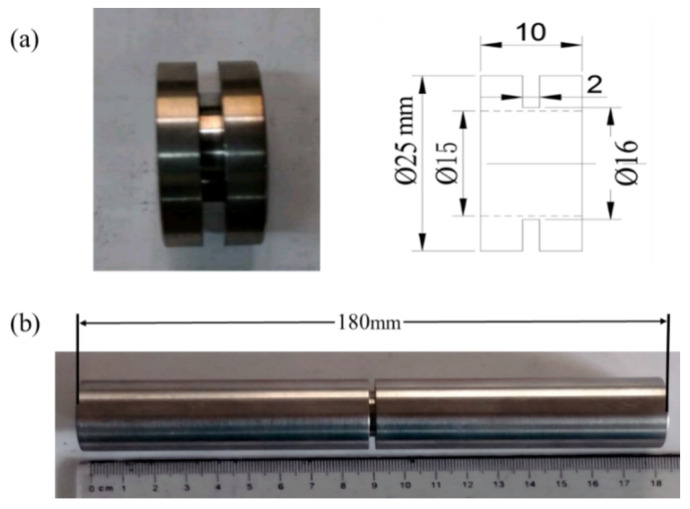
Scheme of the shape and size of TB6 torsional specimen: (**a**) normal-temperature specimen; (**b**) high-temperature specimen.

According to the experimental requirements, the shape of the dynamic shear specimen was designed as a thin-walled circular tube, and the actual length of the experimental section was determined by the strain rate required for the experiment. The overall length of the high-temperature specimen was larger than that of the normal-temperature specimen due to the consideration of the heating and cooling factors. The specific shape and dimensions of the specimen are shown in Figure 4. The outer diameter of the gauge section is *D* = 16 mm, the inner diameter is *d* = 15 mm, and the length is *l* = 2 mm. The outer diameter of the connection between the specimen and the experimental equipment is *R* = 25 mm, the total length of the normal-temperature specimen is *L* = 20 mm, and the total length of the high-temperature specimen is *L* = 180 mm.

Microstructure observation was performed at the center of the sample cross-section using a JEOL-JSM 7001F field emission gun-scanning electron microscope (FEG-SEM) (JOEOL, Tokyo, Japan) operating at 15 kv voltage and 10 mm working distance. A thin 0.3 mm slice was cut along the axial direction of the sample using an electric spark machine. They were mechanically ground to about 0.1 mm thick. Then, thin foils for transmission electron microscopy (TEM) were prepared by electrolysis and particle thinning. TEM was performed on a probe-corrected scanning transmission electron microscope JEOL 2100F (JEOL, Tokyo, Japan) operated at 200 kV and equipped with a cold field emission gun. To identify the present phases, a PANalytical X’pert PRO multipurpose diffractometer (MPD) (PANanalytical, Almelo, The Netherlands) was used. The diffraction pattern from a sample was obtained over the 2θ range of 30–90° under a continuous scanning mode. The step size and the acquisition time were 0.01 and 500 s, respectively. Auto Rietveld was used in XRD analysis.

## 3. Results

### 3.1. Shear Stress-Strain Curves

**Figure 5:**
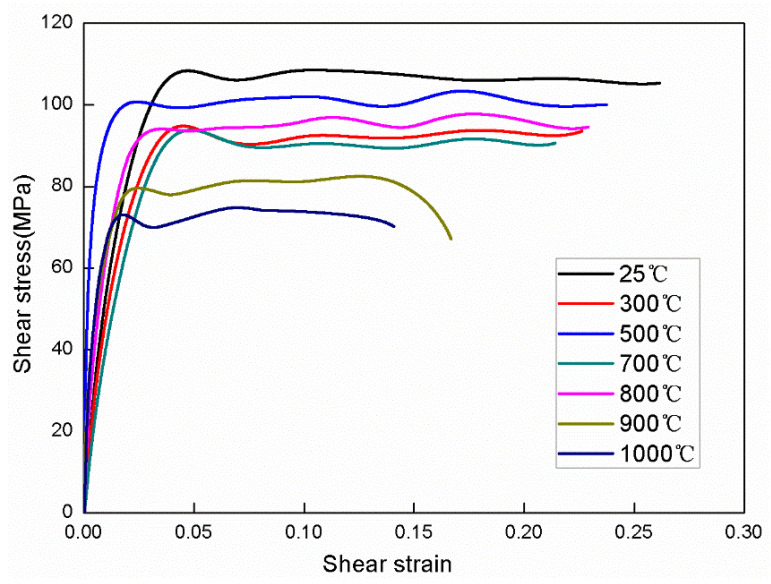
Shear stress-strain curve of Ti-1023 specimen at different temperatures.

Figure 5 shows the shear stress-strain curves of the Ti-1023 alloy after dynamical torsion at different temperatures with a strain rate of 10^3^. It can be seen that the shear-yield stress decreases significantly with the increase of temperature. The plastic flow process is relatively smooth and does not show a significant strengthening effect due to the effect of temperature. However, in the temperature range of 500–700 °C, the shear-yield stress increases with the increase of temperature. When the experimental temperature increases to the material phase transition temperature (about 800 °C), the yield stress decreases with the increase of temperature. The yield strain and failure strain do not show obvious temperature dependence. Figure 5 also indicates that the critical point of failure of Ti-1023 alloy occurs when the strain is 0.28. Beyond this point, the shear stress–strain curve declines slowly, and shear localization begins to occur inside the material to form micro-damage. The experimental results show that the performance of Ti-1023 is relatively good at a temperature around 700 °C, and the enhanced plasticity makes the instability stage last longer without rapid fracture. The abnormal yield stress is related to the evolution of the microstructure after the material is subjected to impact loading, which will be discussed in the following section.

After a number of repeated experiments, we find that there is no significant difference between the behavior of the curves at temperatures of 300 °C, 700 °C, and 800 °C. We believe that the stress-induced martensite phase produced by impact can offset the effect of temperature on the material strength, and the content of α″ phase is significantly higher at 700 °C and 800 °C than that at 300 °C, which to a certain extent, also reflects the excellent high-temperature performance of the alloy.

### 3.2. Microstructural Evolution in the Deformed Region

**Figure 6:**
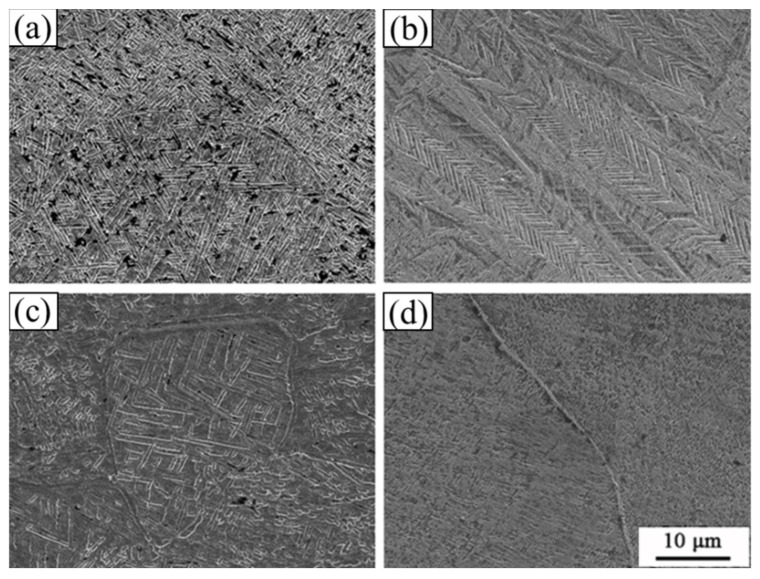
Ti-1023 microstructure of torsional region at different temperatures: (**a**) 300 °C; (**b**) 500 °C; (**c**) 700 °C; (**d**) 900 °C.

The thin-walled cylindrical specimen after the experiment was cut in half along the axial direction with a wire-cutting machine, and the microstructure was observed after grinding, polishing, and corrosion. The etching solution used was a Kroll reagent with the ratio of HF:HNO_3_:H_2_O = 1:2:50. Figure 6 shows the microstructure of the specimens tested at different temperatures. It can be seen that the grain size is significantly refined after the alloy is subjected to torsional shock load at 300 °C. When the temperature reaches 500 °C (Figure 6c), the microstructure presents a sawtooth-shape-like characteristic, which is formed by two groups of innumerable strips arranged in parallel. The sawtooth microstructure distributes in a straight line with some terminating in the grain or grain boundary and some traversing through several grains. This is the typical morphology of the stress-induced martensitic α″ phase created during deformation, which can significantly improve the mechanical properties of Ti-1023 [26,27,28,29].

Figure 6c,d show the microstructure of the alloy after the experiment near the phase transition temperature from which no sawtooth-like structure is found. When the temperature is higher than the phase transition temperature, the α phase transforms into the β phase that is more prone to plastic deformation, and the grain refinement is more obvious, resulting in a decrease in the yield stress of the alloy. Therefore, temperature becomes the main factor affecting the yield strength when the temperature is higher than the phase transition temperature.

**Figure 7:**
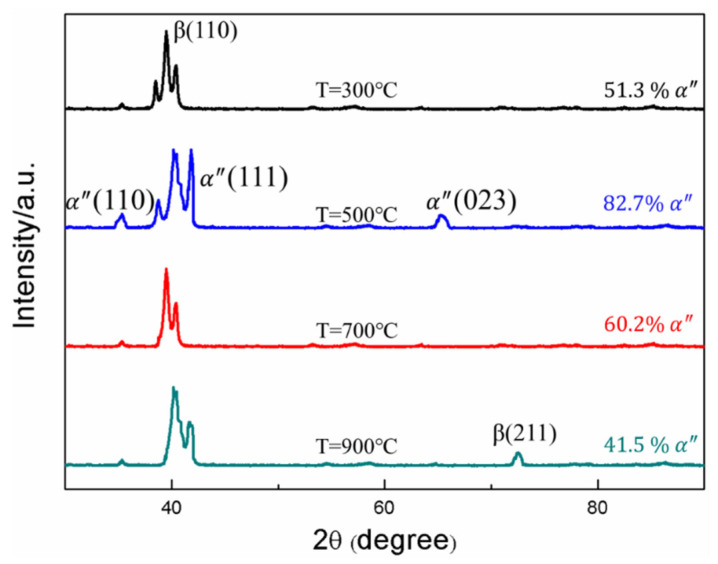
Torsional region X-ray diffraction patterns of TB6 at different temperatures.

The XRD patterns of the specimen subjected to torsional impact load at different temperatures are shown in Figure 7. It can be seen that throughout the high-temperature impact process, the cubic β phase transforms into the orthorhombic martensitic α″ phase, and no other phases are found. The α″ phase peak begins to become stronger, indicating that the volume fraction of the martensite phase increases. With the further increase of temperature, the intensity of the β peak becomes stronger when the temperature is higher than the phase transition temperature (800 °C). This means that part of the α″martensite transforms back to the β phase, and the grains in the structure are refined. Temperature becomes the main factor affecting the strength of the material. Through the statistics of the volume fraction of martensite phase, it is found that the volume fraction is the highest at 500 °C, indicating that the stress-induced martensite has a certain influence on the strength of the material. The reason for the volume fraction of the α″ phase with an increase in temperature was the change in plastic strain. When temperature increases, the degree of deformation of the sample increases, and the plastic strain increases. In a certain range, the volume fraction of α″ phase rises with the increase of temperature. The results of XRD shown that the content of α″ martensite can be controlled by adjusting the impact temperature or strain rate to improve the material properties.

**Figure 8:**
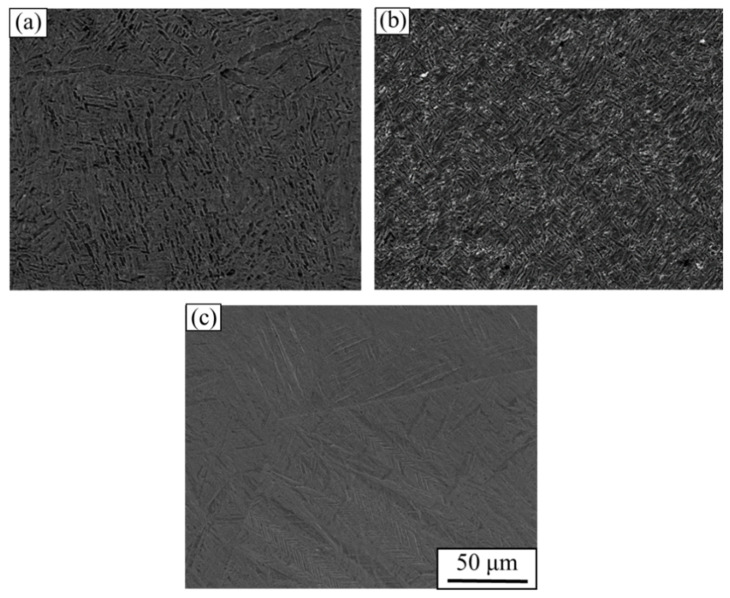
Microstructure of Ti-1023 stress at abnormal temperature: (**a**) original structure; (**b**) structure after 500 °C heating; (**c**) structure after torsional impact at 500 °C.

Figure 8 shows the microstructure of the Ti-1023 alloy at an abnormal stress temperature in which Figure 8a is the original microstructure, Figure 8b is the microstructure after heating the specimen at 500 °C only, and Figure 8c is the microstructure after being subjected to impact torsion loading at 500 °C. When the specimen is only heated, the grains are refined, and the overall microstructure does not change greatly. But after impact loading, the serrated microstructure appears. This suggests that the serrated microstructure is induced by stress rather than temperature, which is in accordance with the results obtained by other scholars [30,31]. Therefore, it is feasible to control the martensite phase in the Ti-1023 alloy to improve its properties by deformation. In addition to the impact-loading method mentioned in the paper, aging treatment and compression (tensile) deformation under different loading rates can also lead to martensitic transformation of this kind of titanium alloy. Aging treatment is mainly applied to heat treatment near the phase transition temperature of titanium alloy to change its structure and improve material properties. The compressive (tensile) deformation is mainly controlled by the loading rate and different strain degrees to induce phase transformation [30,31].

**Figure 9:**
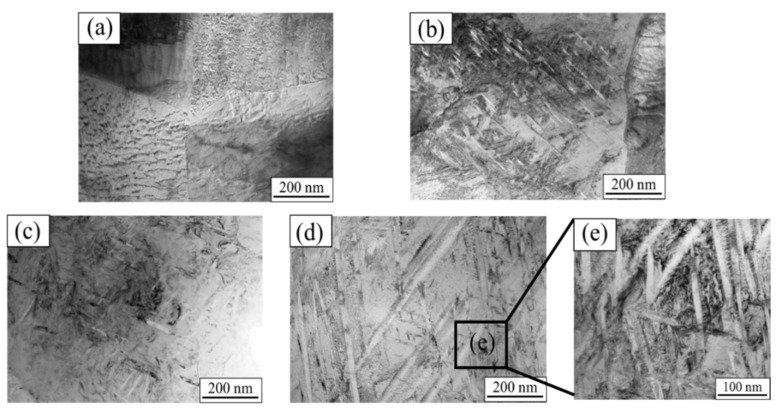
Ti-1023 TEM images of torsional region at different temperatures: (**a**) original tissue; (**b**) 500 °C; (**c**) 700 °C; (**d**) 900 °C; (**e**) figure (**d**) frame-selection magnification graph.

The TEM images of the torsion region of the Ti-1023 alloy are shown in Figure 9. It can be seen from Figure 9a that the original microstructure is mainly composed of dislocations. The needle-like microstructure of the light and dark distribution shown in Figure 9b,c indicates highly elongated dislocation cells that appear after the alloy is subjected to shear loading, and the degree of deformation becomes more severe as the temperature increases based on the observation of the extent of the distribution of the needle-like microstructure. When the temperature is increased to 900 °C, the dislocation density is relatively low, as shown in Figure 9d. And the orientation of martensite will change at 900 °C; in the repeated experiment, the close-packed hexagonal α″ phase was found in very few samples. In addition to elongated dislocation cells, there are also a number of ultra-fine deformed grains and recrystallized grains with a width of about 100 nm (Figure 9e). When titanium alloys undergo severe plastic deformation, shear localization occurs and adiabatic shear bands appear, and DRX grains are finer than the original structure, which can usually be observed in the ASB region. In the current work, a similar structure in the microstructure of the 900 °C impact sample is found, indicating that shear localization occurs inside the sample. Similar results have also been found in the adiabatic shear band of the titanium alloys after impact loading [32]. When abnormal stress occurs at 500 °C (Figure 9b), the volume fraction of the elongated dislocation cells is the least, which also reflects the relatively strong shear resistance of the alloy at this temperature.

## 4. Conclusions

In this paper, a test method for high-temperature and high-strain-rate shear performance of materials based on the modified Hopkinson torsion bar was proposed, and the high-temperature dynamic shear behavior of the Ti-1023 alloy was experimentally studied, obtaining the following conclusions:

1. The modified high-temperature Hopkinson torsion bar can achieve high-temperature testing within 1000 °C. The temperature monitoring of the specimen showed that the heating speed is fast during the experiment, and the temperature gradient of the experimental environment is small, which can ensure the validity of the experimental data.
2. The dynamic shear experiments on Ti-1023 found that when the temperature is lower than the transformation temperature, the stress-induced martensite is the key factor affecting the strength of the material; when the temperature is higher than the transformation temperature, the temperature becomes the main reason.
3. The stress-induced martensite generated in Ti-1023 can effectively improve the strength of the material, and has little effect on the plasticity of the material. Therefore, the material properties can be modified by adjusting the content of stress-induced martensite in Ti-1023. The method is also applicable to other near-β phase titanium alloy materials.

## Figures and Tables

**Table 1:** Chemical compositions of the studied Ti-1023 titanium alloy.

Element	Ti	V	Al	Fe	C	O	N	H
content%	84	10.5	3.14	2.1	0.02	0.01	0.03	0.002

## Data Availability

Not applicable.
